# Data from national health registers as endpoints for the Tromsø Study: Correctness and completeness of stroke diagnoses

**DOI:** 10.1177/14034948211021191

**Published:** 2021-06-14

**Authors:** Torunn Varmdal, Maja-Lisa Løchen, Tom Wilsgaard, Inger Njølstad, Audhild Nyrnes, Sameline Grimsgaard, Ellisiv B. Mathiesen

**Affiliations:** 1 UiT The Arctic University of Norway, Tromsø, Norway; 2 University Hospital of North Norway, Tromsø, Norway; 3 Norwegian University of Science and Technology, Trondheim, Norway

**Keywords:** Cardiovascular diseases, registers, data collection, data quality, quality control

## Abstract

**Aim:**

To assess whether stroke diagnoses in national health registers are sufficiently correct and complete to replace manual collection of endpoint data for the Tromsø Study, a population-based epidemiological study.

**Method:**

Using the Tromsø Study Cardiovascular Disease Register for 2013–2014 as the gold standard, we calculated correctness (defined as positive predictive value, PPV) and completeness (defined as sensitivity) of stroke cases in four different data subsets derived from the Norwegian Patient Register and the Norwegian Stroke Register. We calculated the sensitivity and PPV with 95% confidence intervals (CIs) assuming a normal approximation of the binomial distribution.

**Results:**

In the Norwegian Stroke Register we found a sensitivity of 79.8% (95% CI 74.2–85.4) and a PPV of 97.5% (95% CI 95.1–99.9). In the Norwegian Patient Register the sensitivity was 86.4% (95% CI 81.6–91.1) and the PPV was 84.2% (95% CI 79.2–89.2). The overall highest levels were found in a subset based on a linkage between the Norwegian Stroke Register and the Norwegian Patient Register, with a sensitivity of 88.9% (95% CI 84.5–93.3), and a PPV of 89.3% (95% CI 85.0–93.6).

**Conclusions:**

Data from the Norwegian Patient Register and from the linked data set between the Norwegian Patient Register and the Norwegian Stroke Register had acceptable levels of correctness and completeness to be considered as endpoint sources for the Tromsø Study Cardiovascular Disease Register. The benefits of using data from national registers as endpoints in epidemiological studies must be weighed against the impact of potentially decreased data quality.

## Background

Epidemiological population studies are major contributors to the research community worldwide. To be useful data sources for gaining more knowledge of disease risk factors and outcomes it is essential that such studies include correct, complete, and timely endpoints. The Tromsø Study is a population-based, prospective study consisting of seven surveys (Tromsø 1–7) conducted in the municipality of Tromsø during the period 1974–2016 [1,2]. The Tromsø Study Cardiovascular Disease Register was established to collect information on endpoints, and includes incident fatal and non-fatal cases of stroke, in addition to myocardial infarction, atrial fibrillation, and venous thromboembolism [3]. Data collection was predominantly done through expert review of hospital medical records.

Ascertaining cases through expert review of medical records is considered the gold standard of data collection methods and is widely used in health register validation studies [4]. Consequently, it is to be expected that the Tromsø Study Cardiovascular Disease Register is highly correct and complete. However, manual data collection is very resource intensive as it requires a meticulous and time-consuming effort by trained reviewers. Given that the Tromsø Study is a prospective, ongoing study with no defined end-date, ascertainment of endpoints will be necessary for years, or rather decades, to come. In Norway, the Norwegian Patient Register was established with person-identifiable information in 2008, and the Norwegian Stroke Register followed in 2012. Thus, an opportunity emerged to investigate whether linkage to one or both national registries could, fully or partially, replace today’s manual data collection method. In this study, we investigated and compared the correctness and completeness of hospitalized stroke cases in the Norwegian Patient Register and the Norwegian Stroke Register, using the Tromsø Study Cardiovascular Disease Register as the gold standard.

## Methods

In this study, three independent health registers were compared (Table I).

**Table I.** Description of the Tromsø Study Cardiovascular Disease Register, the Norwegian Patient Register, and the Norwegian Stroke Register.

	Tromsø Study Cardiovascular Disease Register	Norwegian Patient Register	Norwegian Stroke Register
Type of register	Population-based epidemiological register	National administrative health register	National medical quality register
Data collection period	1968 ->	From 2008	From 2012
Regulatory status	Informed consent	Mandatory	Mandatory
Hospitals	One hospital (University Hospital of North Norway)	All Norwegian hospitals	All Norwegian hospitals involved in acute stroke care
Inclusion criteria	All Tromsø Study participants with a fatal or non-fatal stroke, both hospitalized and non-hospitalized. Only incident cases are registered. Acute stroke is defined according to the WHO criteria* and confirmed by diagnostic imaging or autopsy (ICD-10 codes I60, I61, I63, I64). Strokes related to trauma, brain surgery, brain tumor, hematologic disease or following a subarachnoid hemorrhage are excluded.	All hospitalizations and outpatient visits in Norwegian public hospitals and in private hospitals included in the public reimbursement policy.	All Norwegian residents ⩾18 years of age hospitalized with acute stroke according to the WHO criteria* (ICD-10 codes I61, I63, and I64). Patients hospitalized with acute stroke following a traumatic head injury, stroke related to intracranial tumors and ischemic stroke following a subarachnoid hemorrhage are excluded.
Data collection method	Review of hospital medical records by independent reviewers. Review of death certificates and linkage to the Norwegian Cause of Death Registry.	Data extracted from the hospitals’ administrative systems based on a predefined set of rules. Cumulative data are transferred to the registry on a monthly basis.	Data initially registered on paper forms by trained physicians and nurses, who subsequently enter data into the registry by use of a web-based form.
Contents	Date of symptom onset, stroke subtype (ischemic, hemorrhagic or unspecified), diagnostic imaging and/or findings from autopsy, hospitalized/non-hospitalized, date of review and registration, death date.	Demographic and administrative data, dates of admission and discharge, the main and secondary discharge diagnoses (ICD-10), codes for diagnostic and therapeutic procedures.	Demographic data, diagnostic and therapeutic procedures, clinical findings, functional status, risk factors, use of prescribed drugs prior to admission and at discharge from hospital. A 3-month follow-up regarding the patients’ functional status and self-perceived health.

* World Health Organization (WHO) criteria: Rapidly developed clinical signs of focal or global disturbance of cerebral function, lasting more than 24 h or leading to death, with no apparent cause other than of vascular origin.

### The Tromsø Study Cardiovascular Disease Register

All incident stroke cases among Tromsø Study participants are included in the Tromsø Study Cardiovascular Disease Register (hereafter referred to as the Cardiovascular Disease Register), both fatal and non-fatal, and both hospitalized and non-hospitalized. The Tromsø Study participants were linked to the Norwegian Cause of Death Registry and to the discharge diagnosis registry at the University Hospital of North Norway, which is the only hospital in the municipality of Tromsø. The next nearest hospital is located 300 km away, consequently, it is likely that University Hospital of North Norway covers practically all stroke hospitalizations among Tromsø Study participants. To ascertain stroke cases, an endpoint committee consisting of experienced personnel reviewed all medical records with an ICD-10 discharge diagnosis of I60–I69, G45, G46, or G81 [5]. They also performed manual and/or electronic text searches in paper (used until 2001) and digital versions of hospital records for the terms “stroke,” “infarction,” “hemorrhage,” “subarachnoid” (Norwegian or Latin equivalents) in participants with an ICD-10 discharge diagnosis of I20–I25, I46–I48, I50, R96, R98, or R99. Non-hospitalized stroke events were validated through records from general practitioners, nursing homes and/or death certificates, when available. Stroke was defined according to the World Health Organization (WHO) criteria as a focal or global neurological impairment of sudden onset and lasting more than 24 h (or leading to death) and of presumed vascular etiology [6]. Study participants who moved out of the study area were lost to follow-up for non-fatal events. The Cardiovascular Disease Register contains endpoints from 1968 onwards, however, the register is several years behind in data collection due to its resource-intensive data collection methods; case ascertainment was not complete beyond the year 2014 at the time of this analysis. The register contains dates of symptom onset and death, stroke subtype (ischemic, hemorrhagic, subarachnoid hemorrhage, or unspecified stroke), diagnostic imaging, findings from autopsy, whether the patient was hospitalized, and date of review and registration.

### The Norwegian Patient Register

The Norwegian Patient Register is an administrative, national health register covering all hospital activity within somatic and psychiatric care. The register contains person-identifiable information on all hospitalizations and outpatient visits in all public hospitals and in private hospitals included in the public reimbursement policy in Norway since 2008. The register is used as basis for reimbursement to hospitals, hospital activity statistics, waiting list statistics, and for research. The Norwegian Patient Register contains demographic, administrative, and health-related data, such as dates of admission and discharge, the main and secondary diagnoses according to the ICD-10 and codes for diagnostic and therapeutic procedures. Data are automatically extracted from the hospitals’ patient administrative systems based on a predefined set of rules, and cumulative data are transferred to the register on a monthly basis.

### The Norwegian Stroke Register

The Norwegian Stroke Register (hereafter referred to as the Stroke Register) is a national medical quality register established in 2012. All Norwegian hospitals are obliged to enter into the Stroke Register medical data on all residents ⩾ 18 years of age hospitalized with acute stroke according to the WHO criteria (ICD-10 codes I61, I63, and I64). Patients hospitalized with acute stroke following a traumatic head injury, stroke related to intracranial tumors, and ischemic stroke following a subarachnoid hemorrhage are excluded from the Stroke Register. The register contains person-identifiable information on the patients’ functional status before stroke onset, past medical history, the use of prescription drugs prior to admission and at discharge, clinical findings on admission to hospital, and diagnostic procedures and treatment received during hospitalization. Furthermore, the register contains a 3-month follow-up of the patients’ functional status and self-reported quality of life. Data are initially registered on paper forms locally at the hospitals by trained physicians and nurses, who subsequently enter data into the Stroke Register by use of a web-based form.

#### Study population

**Figure 1.**
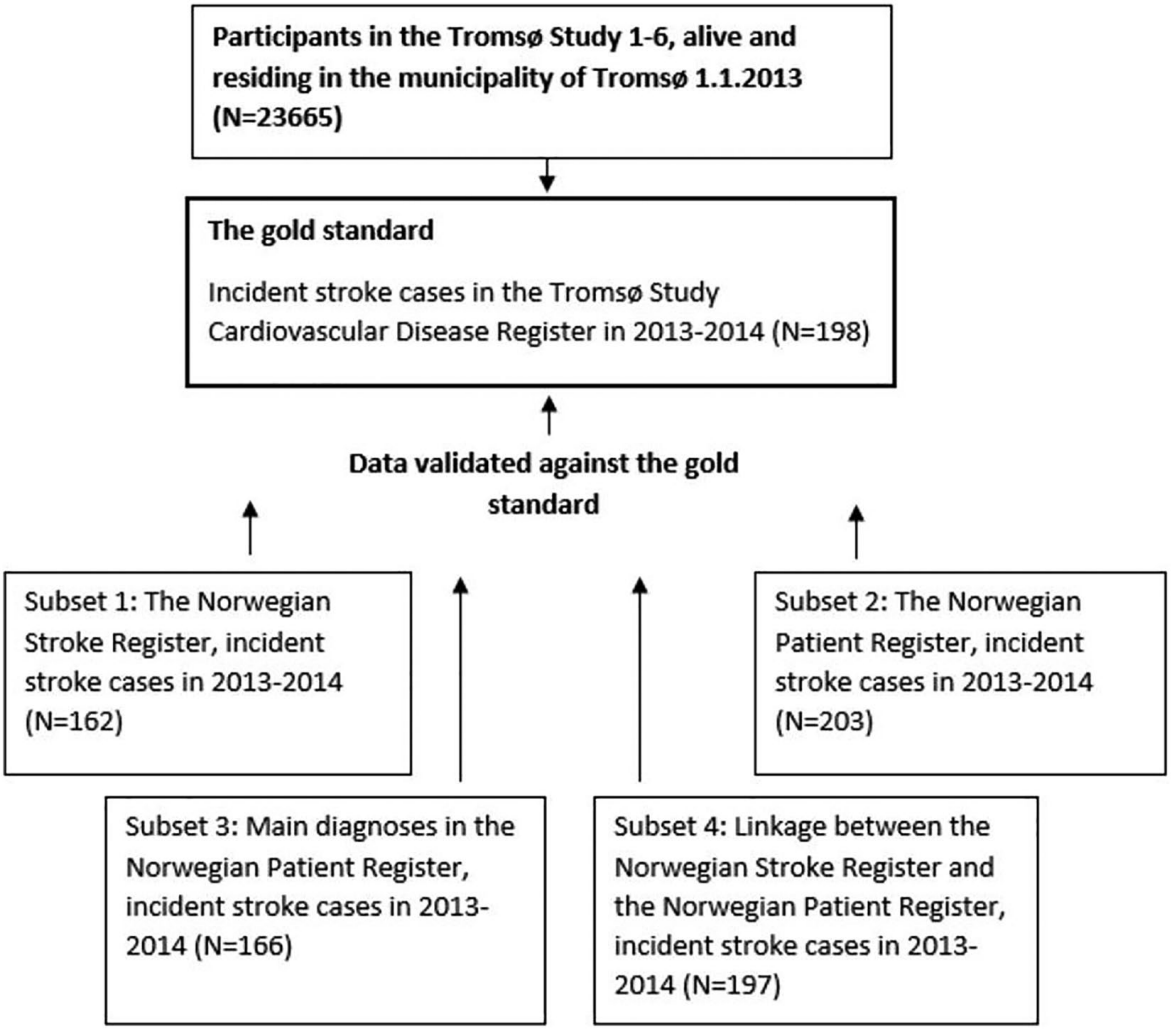
Study population. Validating four subsets of national health registers against the Tromsø Study Cardiovascular Disease Register (the gold standard).

The study population consisted of all participants in the Tromsø Studies 1 through 6 from 1974 to 2008, alive and residing in the municipality of Tromsø by 1 January 2013 (*N* = 23,665). We defined the gold standard as follows: all incident, hospitalized stroke cases occurring in 2013–2014 and registered as “definite stroke” in the Cardiovascular Disease Register (Figure 1). To enable comparison with the Norwegian Patient Register and the Stroke Register, we excluded cases of non-hospitalized stroke (both fatal and non-fatal, *n* = 13). We also excluded cases of subarachnoid hemorrhage, as this diagnosis is not included in the Stroke Register. A total of 198 stroke cases were identified in the gold standard (176 ischemic and 22 hemorrhagic). The registers were linked using the unique identification number issued by the National Population Register to all residents of Norway, and hospitalizations occurring within 28 days of each other were defined as the same cases.

We defined four different subsets of data from the national registers:

Subset 1: In the Stroke Register, we identified incident stroke cases defined as the first hospitalization with a stroke diagnosis in 2013–2014 among patients who had participated in the Tromsø Study at least once and were still living in the municipality. Cases registered as recurrent stroke were excluded. A total of 162 cases were identified (142 ischemic, 19 hemorrhagic and 1 unclassified).
Subset 2: We identified incident stroke cases in the Norwegian Patient Register defined as the first hospitalization during 2013–2014 with a main or secondary diagnosis of stroke (ICD-10 codes I61, I63, or I64). Only patients who had participated in the Tromsø Study at least once and still living in the municipality of Tromsø were included. Patients registered with a stroke diagnosis in the period 2008–2012 were excluded. A total of 203 stroke cases were identified (176 ischemic, 25 hemorrhagic and 2 unclassified).
Subset 3: Similar to Subset 2, except that secondary diagnosis of stroke was excluded. A total of 166 stroke cases were identified (146 ischemic, 18 hemorrhagic and 2 unclassified).
Subset 4: A linkage between the Stroke Register and the Norwegian Patient Register was performed, according to the following definition: *All identified incident stroke cases in the Stroke Register + all identified incident main diagnosis of stroke in the Norwegian Patient Register*. A total of 197 stroke cases were identified (173 ischemic, 22 hemorrhagic and 2 unclassified).

#### Statistical analysis

We compared four subsets of data to the gold standard in the Cardiovascular Disease Register: the Stroke Register, main and secondary diagnosis in the Norwegian Patient Register, only main diagnoses in the Norwegian Patient Register, and a linkage between the Norwegian Patient Register and the Stroke Register.

Based on the established gold standard, we classified cases in the four subsets as true positives (TPs), true negatives (TNs), false positives (FPs) or false negatives (FNs). We defined data completeness as equivalent to the sensitivity (TP/(TP+FN)), that is, the proportion of cases of true stroke according to the gold standard that were also present in the registers. We defined data correctness as equivalent to the positive predictive value (PPV) (TP/(TP+FP)), that is, the proportion of stroke cases present in the registers that were cases of true stroke according to the gold standard [7]. The 95% confidence intervals (CIs) were calculated assuming a normal approximation of the binomial distribution.

All statistical analyses were performed using IBM SPSS Statistics version 26.

## Results

Table II describes the distribution of TPs and FPs and TNs and FNs in the four subsets of data compared with the gold standard.

**Table II.** Distribution of true and false positives and negatives in the four subsets of data compared to the gold standard (the Tromsø Study Cardiovascular Disease Register).

	Gold standard: the Tromsø Study Cardiovascular Disease Register		
	Stroke	No stroke	Total
Subset 1: the Norwegian Stroke Register			
Stroke	158	4	162
No stroke	40	23,463	23,503
Total	198	23,467	23,665
Subset 2: the Norwegian Patient Register, main and secondary diagnoses			
Stroke	171	32	203
No stroke	27	23,435	23,462
Total	198	23,467	23,665
Subset 3: the Norwegian Patient Register, main diagnoses			
Stroke	146	20	166
No stroke	52	23,447	23,499
Total	198	23,467	23,665
Subset 4: linkage between the Norwegian Stroke Register and main diagnoses in the Norwegian Patient Register			
Stroke	176	21	197
No stroke	22	23,446	23,468
Total	198	23,467	23,665

**Figure 2.**
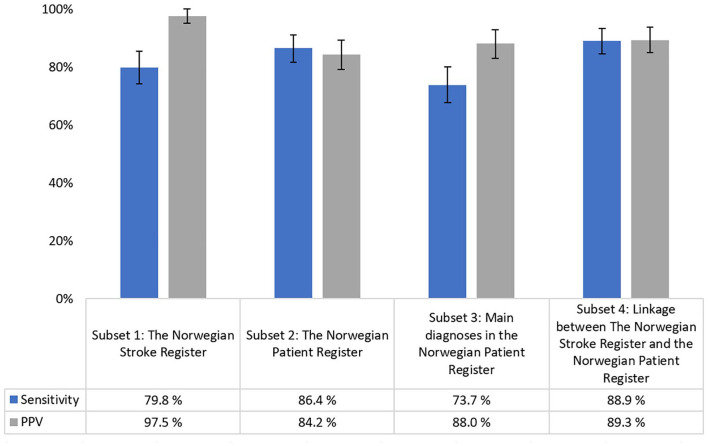
Estimated sensitivity and positive predictive value (PPV) for four different subsets of data compared to the gold standard (the Tromsø Study Cardiovascular Disease Register). Error bars show 95% confidence intervals.

Subset 1: Estimated measures of completeness and correctness of the Stroke Register indicated a sensitivity of 79.8% (95% CI 74.2–85.4%), and a PPV of 97.5% (95% CI 95.1–99.9%) (Figure 2). Among the 40 FN cases, we found that 31 cases were registered with stroke in the Norwegian Patient Register. Three of the four FP cases were admitted to hospitals outside of the municipality of Tromsø.
Subset 2: In the Norwegian Patient Register, estimated sensitivity of incident stroke cases was 86.4% (95% CI 81.6–91.1%) and PPV was 84.2% (95% CI 79.2–89.2%). Of the 32 FP cases, 12 were registered with secondary diagnoses of stroke in the Norwegian Patient Register and were not registered in the Stroke Register. Among the remaining 20 cases, 10 had hospital admittance and discharge on the same dates. Three of the cases had been admitted to hospitals other than the University Hospital of North Norway. Of the 27 FN cases, 11 cases were registered in the Stroke Register. Furthermore, seven cases were registered in the Norwegian Patient Register on different dates than in the gold standard (dates differing by >28 days).
Subset 3: Excluding secondary diagnoses in the Norwegian Patient Register, estimated sensitivity was 73.7% (95% CI 67.6–79.9%) and PPV was 88.0% (95% CI 83.0–92.9%). This analysis resulted in 52 FN cases, of which 33 cases would have been identified if we had not excluded secondary diagnosis of stroke.
Subset 4: Linkage between the Stroke Register and main diagnoses in the Norwegian Patient Register generated an estimated sensitivity of 88.9% (95% CI 84.5–93.3%), and a PPV of 89.3% (95% CI 85.0–93.6%). Of the 21 FP cases, 10 were admitted and discharged from hospital on the same date, and two were admitted to hospitals other than the University Hospital of North Norway. Of the 22 FN cases, four were registered in the Norwegian Patient Register on different dates than in the gold standard (dates differing by >28 days), and eight would have been identified as stroke cases if we had included the secondary diagnosis of stroke.

## Discussion

The aim of this study was to investigate whether linkage with national registers can replace the current manual data collection method in an epidemiological study: the Tromsø Study Cardiovascular Disease Register. We chose to validate data completeness and correctness of four different data subsets from the national registers, as we hypothesized that the different subsets would generate different results.

The Stroke Register was moderately complete (sensitivity 79.8%) and highly correct (PPV 97.5%). A probable cause for the moderate completeness was the registers’ low coverage for the year 2013. Established in 2012, the Stroke Register had a national coverage of 63% and 87% in 2013 and 2014, respectively. Consequently, an investigation of more recent data may result in a higher degree of completeness. The near perfect correctness can be explained by the Stroke Register’s data collection method. Data are manually validated by trained personnel, thus minimizing the probability of entering false stroke cases into the register.

A previous study validated stroke diagnoses in the Norwegian Patient Register compared with a review of medical records and found the register to be highly complete but moderately correct [8]. The study pointed out that approximately 50% of the secondary diagnoses of stroke in the register were FPs, mainly due to cases of stroke sequelae being incorrectly registered as acute stroke in the hospital administrative systems. Consequently, we chose to split the validation of the Norwegian Patient Register into two subsets: first, we investigated the main and secondary diagnoses combined, and second, we investigated only the main diagnoses of stroke.

We found a high degree of completeness and correctness in the Norwegian Patient Register when we studied main and secondary diagnoses combined (sensitivity 86.4% and PPV 84.2%). We identified 32 FP cases of which 12 had a secondary diagnosis of stroke, none of which were registered in the Stroke Register. Combined with the knowledge from the aforementioned validation study, our findings suggest that some of these were not true stroke cases. Furthermore, 10 cases were registered with hospital admittance and discharge with the same dates, thus it is questionable whether all these cases were true stroke cases, as acute stroke hospitalizations with a length of stay 0–1 days rarely occur.

As expected, correctness was higher (PPV 88.0%) when we excluded the secondary diagnoses of stroke from the analyses. However, higher correctness came at a cost of lower completeness (sensitivity 73.7%). Excluding the secondary diagnoses implied losing some true stroke cases, while including them would lead to adding some false stroke cases.

The Norwegian Institute of Public Health annually links the Stroke Register and the Norwegian Patient Register for the purpose of calculating the coverage of the registers, based on an assumption that a combination of the two registers yields the most complete record of hospitalized stroke cases available in the national registers in Norway. For the same reason, we analyzed a similarly linked subset consisting of all stroke cases in the Stroke Register and main diagnoses of stroke in the Norwegian Patient Register. The results indicated a high degree of completeness and correctness, with an estimated sensitivity of 88.9% and PPV of 89.3%. In the linked data set, including secondary diagnoses of stroke would have resulted in higher completeness, but lower correctness.

We compared three different types of health registers: an epidemiological register, an administrative register, and a medical quality register. Being of different type and origin, the three registers differ from each other in terms of inclusion criteria, data collection methods, and contents. Our study highlighted the pros and cons of each data source. Manual data collection, as in the Stroke Register, secures a high degree of correctness due to assessment of all cases by experts before reporting to the register. At the same time, manual data collection is time-consuming and is likely to negatively affect the register’s completeness. Automatic data extraction from hospital administrative systems, conversely, can often be beneficial for data completeness while potentially negative for data correctness. A report from The Office of the Auditor General of Norway pointed out that poor quality medical coding in hospitals leads to errors in the health statistics [9].

Previous studies [10–14] including a recent systematic review [4] have investigated the validity of stroke diagnoses in hospital discharge registers, administrative health registers, and medical quality registers. Their results indicated sensitivity in the range 70–90% and PPV in the range 60–100%, placing our results well within these ranges. However, differences in sample attributes, sample sizes, and methods of validation make direct comparison between studies difficult.

No clear and unambiguous cut-off for what is considered “moderate” and “high” data quality exists, yet most authors of previous studies seem to agree that sensitivity or PPV ⩾80% can be considered fairly good/good and ⩾90% can be considered very good/excellent. Inherently, these cut-offs are arbitrary and relative and must be interpreted in light of the intended use of the data in question. Collecting endpoint data from national registers rather than from hospital medical records will enable easy access to updated stroke endpoints in the Cardiovascular Disease Register. Our results indicated that this will probably result in somewhat less complete and correct endpoints for the register. However, two of our four subset analyses showed acceptable levels of completeness and correctness: Subset 2 the Norwegian Patient Register (sensitivity and PPV in the range 84–86%) and Subset 4 linkage between the Stroke Register and the Norwegian Patient Register (sensitivity and PPV approximately 89%).

Our results illustrated the importance of assessing completeness and correctness in relation to each other when comparing different types of health registers [15]. The intended purpose of use determines which data source is the most suitable. Often, compromises are necessary to achieve the desired balance between completeness and correctness.

In this study, we had access to person-identifiable data sets from three health registers, thus allowing for identification and linkage of each unique stroke case across the data sets. Another strength of the study was the comparison between different types of registers, which highlights the importance of data quality awareness when using data from different types of health registers. The main limitation of this study was the inability to unambiguously identify incident stroke cases in the Stroke Register and the Norwegian Patient Register. The Norwegian Patient Register includes data from 2008, thus leaving the possibility of false identification of an incident stroke in 2013–2014 in cases where a patient had a stroke prior to 2008. The Stroke Register, conversely, contains a specific variable for whether the stroke was incident or recurrent. We used the information in this variable when identifying incident stroke cases, but we do not have any information on the quality of the variable.

Importantly, our study only investigated the hospitalized stroke cases, as the national registers exclude non-hospitalized cases. In the Cardiovascular Disease Register, we identified 13 non-hospitalized stroke cases in 2013–2014. In the event of conversion from manual data collection to linkage with national registers, fatal non-hospitalized cases can be collected from the Norwegian Cause of Death Registry. The impact of missing the non-fatal non-hospitalized cases of stroke in the Cardiovascular Disease Register will be negligible, as these cases are rare.

## Conclusion

This study highlights the data quality aspects of different types of health registers and demonstrates that the pros and cons of each register type are often reflected in their data quality. For the Tromsø Study Cardiovascular Disease Register, we found that two of the national register subsets had acceptable levels of correctness and completeness to be considered as endpoint sources: the Norwegian Patient Register and a linkage between the Stroke Register and main diagnosis in the Norwegian Patient Register. The benefits of using data from national registers as endpoints in epidemiological studies include faster, less resource-intensive access to nationwide data, as opposed to manual data collection methods. These benefits must be weighed against the impact of potentially decreased data quality.
